# Associations of systemic immune‐inflammation index with high risk for prostate cancer in middle‐aged and older US males: A population‐based study

**DOI:** 10.1002/iid3.1327

**Published:** 2024-06-24

**Authors:** Wentao Yao, Jiacheng Wu, Ying Kong, Feng Xu, Yinyi Zhou, Qing Sun, Qingqing Gao, Zhenyu Cai, Chendi Yang, Yuhua Huang

**Affiliations:** ^1^ Department of Urology The First Affiliated Hospital of Soochow University China; ^2^ Department of Urology Suzhou TCM Hospital Affiliated to Nanjing University of Chinese Medicine China; ^3^ Department of Urology Affiliated Tumor Hospital of Nantong University & Nantong Tumor Hospital China; ^4^ Department of Preventive Medicine Suzhou TCM Hospital Affiliated to Nanjing University of Chinese Medicine China

**Keywords:** middle‐aged and older males, NHANES, prostate cancer, prostate‐specific antigen, systemic immune‐inflammation index

## Abstract

**Background:**

Systemic immune‐inflammation index (SII) provides convincing evaluation of systemic immune and inflammatory condition in human body. Its correlation with prostate cancer (PCa) risk remains uncharted. The principal objective of this investigation was to elucidate the association between SII and the risk for PCa in middle‐aged and elderly males.

**Materials and Methods:**

Analysis entailed multivariate linear and logistic regression, generalized additive model, and smoothing curve fitting using resource from 2007 to 2010 National Health and Nutrition Examination Survey (NHANES). To ascertain robustness and consistency of this association across different demographic strata, we conducted rigorous subgroup analyses and interaction tests.

**Results:**

Among 3359 participants, those with elevated SII displayed higher total prostate‐specific antigen (tPSA) levels, higher risk for PCa, and lower free/total PSA (f/t PSA) ratio. Specifically, each unit increase of log_2_ (SII) was associated with a 0.22 ng/mL increase in tPSA (β: 0.22, 95% confidence intervals [CI] 0.05–0.38), a 2.22% decline in f/t PSA ratio (β: −2.22, 95% CI −3.20 to −1.23), and a 52% increased odds of being at high risk for PCa (odds ratio [OR]: 1.52, 95% CI 1.13–2.04). People in the top quartile of log_2_ (SII) exhibited 0.55 ng/mL increased tPSA (β: 0.55, 95% CI 0.19–0.90), 4.39% reduced f/t PSA ratio (β: −4.39, 95% CI −6.50 to −2.27), and 168% increased odds of being at high risk for PCa (OR: 2.68, 95% CI 1.32–5.46) compared to those in the bottom quartile.

**Conclusion:**

Systemic immune and inflammatory condition, as represented by SII, is independently and positively associated with tPSA levels and the risk for PCa, as well as independently and negatively associated with f/t PSA ratio among middle‐aged and older US males. These findings may enhance the effectiveness of PCa screening in predicting positive biopsy results.

## 1 INTRODUCTION

Prostate cancer (PCa) stands as the most prevalent malignancy diagnosed in men, and it ranks second in terms of cancer‐related mortality in men within the United States. In 2023, a total of 288,300 newly diagnosed PCa cases and 34,700 PCa‐related deaths were expected.^1^ Alarmingly, the PCa incidence rate increased by 3% annually from 2014 through 2019, concomitant with a notable shift toward higher grades and stages of diagnosis.^2^

The protein secreted by prostatic epithelial cells, known as prostate‐specific antigen (PSA), was introduced to PCa screening in 1994.^3^ The widespread adoption of PSA testing has undeniably enhanced the detection of early‐stage PCa and conferred survival advantages upon patients. Nonetheless, the diagnostic efficacy of PSA screening has been challenged by an array of factors, and its low specificity has sparked considerable debate within the medical community about overdiagnosis and overtreatment.^4^ Consequently, the quest for risk factors linked to PCa assumes paramount clinical significance in ensuring the quality of disease screening.

It is believed that the formation and metastasis of tumors are all influenced by the interaction between immune response and systemic inflammation.^5^ Likewise, inflammation has been proven to be a major contributor to the prostatic carcinogenesis and progression from different methodological perspectives, such as genetic, epidemiological, and molecular pathological study methods.^6^ Current research primarily explores the impact of local chronic inflammation, such as prostatitis, on the risk of developing PCa.^7^ Although existing epidemiological evidence indicates a strong association between systemic inflammation and unfavorable prognosis in PCa patients,^8^ the link between systemic inflammation and PCa risk is poorly defined. Given the close association of inflammation with PCa, further investigating the PCa risk using systemic inflammatory markers across adults without a PCa history is of great significance.

Inflammation associated with cancer involves a variety of components such as immune cells, cytokines, and protein inflammation mediators. The primary immune cells in peripheral blood are neutrophils, lymphocytes, platelets, and monocytes. Recently, parameters, combining these inflammatory cells, including platelet‐lymphocyte ratio (PLR), neutrophil‐lymphocyte ratio (NLR), and lymphocyte‐monocyte ratio (LMR) have been recognized as clinical indicators of inflammation in research investigating the relationship between inflammation and PCa.^9^ A prospective study reported PLR having higher accuracy in predicting an initial diagnosis of clinically significant PCa (csPCa) than NLR.^10^ In a cross‐sectional study utilizing the National Health and Nutrition Examination Survey (NHANES) cohorts, McDonald et al. observed that NLR was positively associated with elevated total PSA (tPSA), whereas PLR did not demonstrate this association.^11^ However, whether men with elevated NLR are at higher PCa risk was not examined in this study. Additionally, a meta‐analysis confirmed a strong association between LMR and the prognosis of patients with PCa.^12^

Incorporating these parameters, the systemic immune‐inflammation index (SII) was initially employed in 2014 to gauge the prognosis of patients afflicted with hepatocellular carcinoma.^13^ SII is now considered to be a more comprehensive marker of the immune response and inflammatory status in human body and has been investigated widely.^14^ Recent studies have proposed the SII as an indicator of cancer‐related inflammation and a prognostic marker for unfavorable outcomes in PCa.^15^ Furthermore, a retrospective study demonstrated that SII owned a superior predictive value over PLR, NLR, and LMR in patients with localized PCa.^16^ Nevertheless, there is no data on how SII links to PCa risk among men without clinical PCa. This is relevant, because if SII can be shown to track with PCa risk, it may become a valuable supplementary tool for PCa screening. Moreover, SII is an easily measurable and cost‐effective parameter, readily calculated from complete blood counts.

Elevated serum PSA concentration was known to be closely related to the progression of PCa. In addition, f/t PSA ratio was harnessed as a complementary tool for PCa early diagnosis, especially in cases where tPSA levels fall within the gray zone (4 ng/mL to 10 ng/mL).^17^ The prostate health index (PHI), calculated from tPSA, free PSA (fPSA), and the PSA isoform [−2]proPSA, was also approved by the FDA for further testing in patients with PSA levels in the gray zone.^18^ A meta‐analysis indicated that, compared to f/t PSA ratio, using PHI significantly increases the likelihood of detecting positive biopsies in men with PSA levels of 2–10 ng/mL.^19^ Moreover, a prospective study demonstrated that a neural network analysis combining PHI and mpMRI significantly improves the identification of pathological csPCa in those with PSA between 2 and 10 ng/mL, aiding in better preoperative risk stratification.^20^ However, the pre‐analytical instability and high cost of [−2]proPSA limited the clinical application of PHI.^21^ Accordingly, the f/t PSA ratio continues to be the predominant biomarker test for further assessing cancer risk before biopsy in subjects with PSA values in the gray zone. Hence, our investigation was undertaken to uncover SII and the risk of PCa correlation, leveraging pertinent PSA laboratory data, including tPSA, fPSA, and f/t PSA ratio, extracted from 2007 to 2010 NHANES.

## 2 MATERIALS AND METHODS

### 2.1 Study population

**Figure 1:**
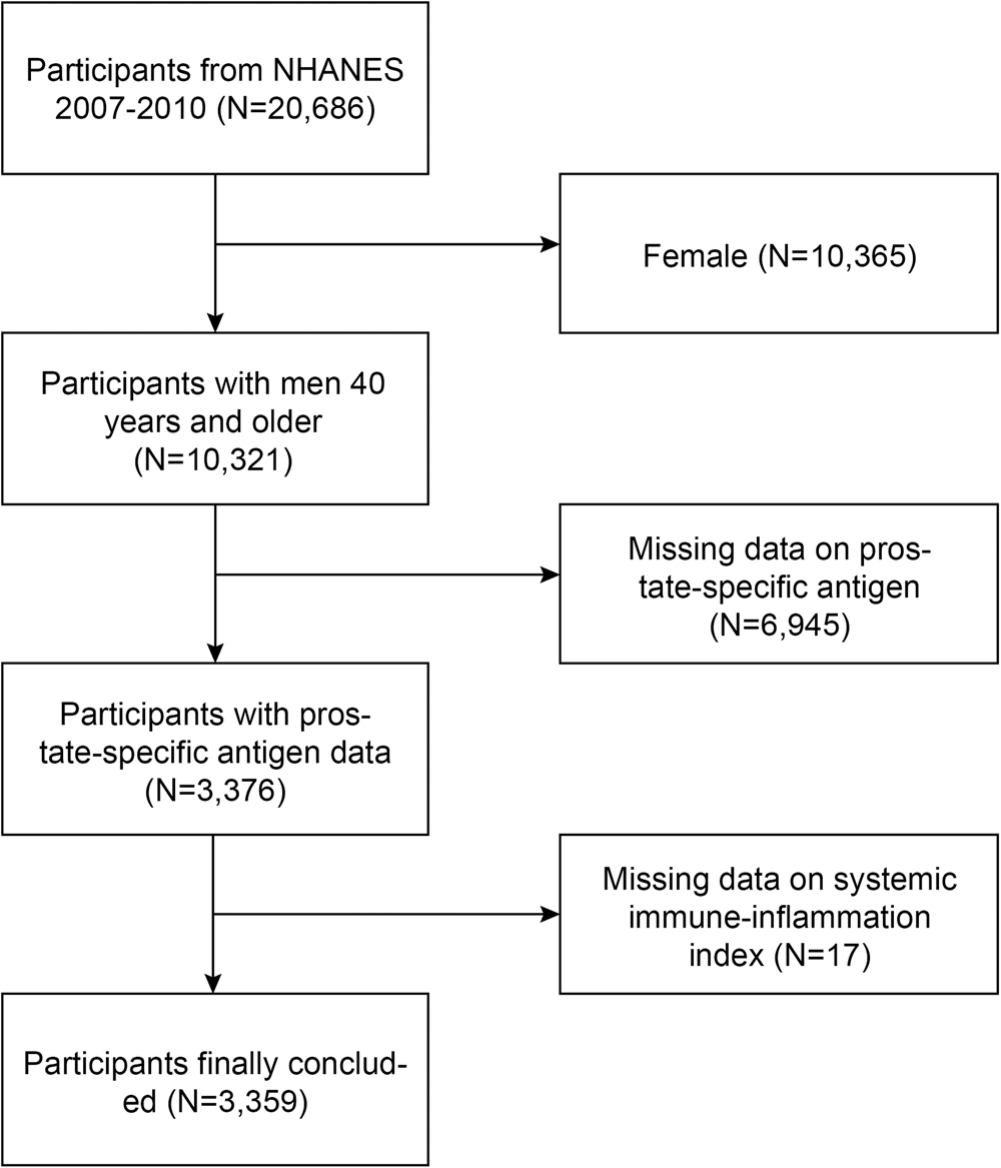
Subjects selection workflow. NHANES, National Health and Nutrition Examination Survey.

Information was acquired from two survey cycles conducted during 2007–2010 as the basis for this cross‐sectional study. All information was NHANES‐sourced, a comprehensive and nationally representative survey dedicated to monitoring US population health & nutritional profiles. The combined application laboratory and physical examinations and interviews is a unique feature for NHANES. Before the commencement of the survey, all the subjects provided informed written consent, and protocol was sourced from the Research Ethics Review Board of the National Center for Health Statistics (NCHS). Comprehensive details of this nationwide survey can be accessed at https://www.cdc.gov/nchs/nhanes/ without application. A total of 3359 participants remained for further analysis after excluding female participants (*N* = 10,365), participants without available PSA data (*N* = 6945), and participants with missing or incomplete SII data (*N* = 17) (Figure 1).

### 2.2 SII

The Beckman Coulter MAXM instrument was employed for the enumeration of platelet, neutrophil, and lymphocyte counts. The SII computation employs the subsequent formula: SII =  platelet count × neutrophil count/lymphocyte count, offering a reliable estimation of surveyed individuals' inflammatory status. As the SII was found to be right‐skewed distributed, it was then log_2_‐transformed in further analysis (Figure S1).^22^

### 2.3 PSA levels and risk for PCa

Male participants aged ≥40 years and without a recent prostate biopsy, rectal examination, cystoscopy, current prostate infection or inflammation, or PCa history were tested for PSA. Blood specimens were obtained from subjects and PSA concentrations were detected by immuno‐enzymatic “sandwich” assay. The risk for PCa is indicated by tPSA and f/t PSA ratio. Specifically, high‐PCa‐risk subjects were defined as those with tPSA >10 ng/mL or tPSA 4–10 ng/mL but f/t PSA ratio ≤25%; the remaining participants were classified as low‐PCa‐risk cohort.^18^, ^23^

### 2.4 Covariables

To address potential confounding effects, the following variables were integrated into the adjustments: age (years), race (Non‐Hispanic White, Non‐Hispanic Black, or other races), education level (below high school, at high school level, or exceeding high school), poverty (yes or no), living status (with partners or alone), smoking status (Nonsmoker, former smoker, or current smoker), drinking status (consuming ≥12 or <12 drinks annually), work activity (inactive, moderate, or vigorous), physical activity (inactive, moderate, or vigorous), hypertension (yes or no), diabetes (yes or no), body mass index (BMI, kg/m²), waist circumference (cm), triglyceride (mg/dL), low‐density lipoprotein cholesterol (LDL‐C)/total cholesterol/high‐density lipoprotein cholesterol (HDL‐C) (mg/dL), C‐reactive protein (CRP, mg/dL), and vitamin D (nmol/L). Specifically, (1) poverty income ratio (PIR) < 1 was used as a cutoff value for poverty^24^; (2) smoking status was categorized based on two questions: “Consumed a minimum of 100 cigarettes during your lifetime?” and “Are you currently a cigarette smoker?”^25^; (3) work activity grouping was established based on two questions: “Does your occupation entail high‐intensity activities that result in significant elevations in respiration or heart rate?” and “Is your job associated with moderate‐intensity tasks leading to minor elevations in respiration or heart rate?”; (4) physical activity classification was created based on two questions: “Do you do any high‐intensity sports, fitness, or recreational activities that result in significant elevations in respiration or heart rate?” and “Do you do any moderate‐intensity sports, fitness, or recreational activities leading to minor elevations in respiration or heart rate?”^26^; (5) hypertension was discerned based on participants' self‐declaration, antihypertensive medication use, or average value of systolic pressure measured four times ≥130 mmHg and/or average value of diastolic pressure measured four times ≥80 mmHg^27^; (6) diabetes determination relied upon participants' own acknowledgment, reliance on antidiabetic medications or insulin, an elevated glycohemoglobin level ≥6.5%, or a fasting glucose reading ≥7.0 mmol/L.^28^

### 2.5 Statistical analysis

The study delved into the demographic profiles of participants categorized by quartiles of the SII via the utilization of chi‐square tests and *t*‐tests. The relationship between SII and PCa risk was comprehensively explored through diverse analytical avenues. Multiple linear regression models were deployed to examine the associations involving PSA levels, encompassing tPSA, fPSA, and the f/t PSA ratios. To assess the risk for PCa, multiple logistic regression models were adopted. Following the transformation of SII from a continuous variable to categorical, a trend test was employed to delineate the linear trends in the relationship between SII and PCa risk. Furthermore, the investigation delved into potential nonlinear associations through smoothing curve fitting and the application of a generalized additive model. To ensure the robustness and consistency of the relationship between SII and PCa risk across diverse subgroups, subgroup analyses and interaction tests were conducted. The analytical processes were executed using R (version 4.2) and EmpowerStats (version 4.1). Statistical significance: *P*‐value below 0.05.

## 3 RESULTS

### 3.1 Participant characteristics

Weighted distribution of baseline characteristics is presented in Table 1, both overall and by quartile of log_2_ (SII). The 3,359 subjects average age was 55.86 ± 11.15 years, with 75.06% of Whites. The mean tPSA, fPSA, and f/t PSA ratio for all adults were 1.50 ± 2.36 ng/mL, 0.37 ± 0.45 ng/mL, and 30.71 ± 12.32, respectively. The average log_2_ (SII) was 8.89 ± 0.81, with an interquartile distribution of Q1: <8.3910; Q2: 8.3911–8.8772; Q3: 8.8773–9.3792; Q4: >9.3793. The prevalence of high PCa risk across different SII levels was reported as follows: 3.49%, 4.15%, 3.54%, 8.73%, respectively. Compared with the lowest SII quartile, top‐quartile people are more prone to being of an older age, Caucasian ethnicity, residing alone, being active smokers, abstaining from alcohol consumption, engaging in low levels of physical activity, and experiencing a heightened risk of PCa (*p* < 0.05). Besides, they had higher waist circumference, LDL‐C, CRP, HDL‐C, BMI, vitamin D, total cholesterol, tPSA, and fPSA, as well as lower f/t PSA ratio (*p* < 0.05).

**Table 1:** Study population baseline features.

	log_2_ (SII)					*P*‐value
Characteristics	Overall (*N* = 3359)	Q1 (*N* = 840)	Q2 (*N* = 839)	Q3 (*N* = 840)	Q4 (*N* = 840)	
Age (years)	55.86 ± 11.15	55.07 ± 10.75	54.91 ± 10.88	55.62 ± 11.26	57.82 ± 11.46	<0.001
**Race/ethnicity, (%)**						<0.001
Non‐hispanic white	75.06	67.94	74.61	78.80	78.17	
Non‐hispanic black	8.69	14.63	9.24	5.17	6.33	
Other races	16.26	17.42	16.15	16.03	15.50	
**Education level, (%)**						0.119
<High school	19.64	21.39	16.77	18.14	22.52	
High school	24.82	23.51	24.79	26.00	24.82	
>High school	55.52	55.10	58.39	55.86	52.66	
**Poverty, (%)**						0.629
Yes	9.36	10.59	8.77	9.11	9.08	
No	90.64	89.41	91.23	90.89	90.92	
**Living status, (%)**						<0.001
With partners	75.53	75.77	80.14	77.28	68.74	
Alone	24.43	24.23	19.80	22.72	31.18	
**Smoking status, (%)**						0.011
Nonsmoker	44.19	48.59	44.26	44.78	39.28	
Former smoker	35.46	32.65	37.66	34.44	37.07	
Current smoker	20.31	18.66	18.08	20.76	23.66	
**Drinking status, (%)**						<0.001
≥12 drinks/year	85.36	85.31	87.63	88.23	79.90	
<12 drinks/year	14.58	14.69	12.26	11.72	20.02	
**Work activity, (%)**						0.118
Inactive	50.13	50.55	48.67	50.69	50.59	
Moderate	22.96	25.73	22.02	21.17	23.23	
Vigorous	26.91	23.71	29.31	28.13	26.18	
**Physical activity, (%)**						0.021
Inactive	50.32	47.88	48.09	49.49	55.83	
Moderate	29.13	29.54	30.37	29.93	26.62	
Vigorous	20.54	22.58	21.54	20.58	17.55	
**Hypertension, (%)**						0.252
Yes	60.76	59.25	58.92	63.09	61.48	
No	39.24	40.75	41.08	36.91	38.52	
**Diabetes, (%)**						0.099
Yes	17.98	19.32	16.42	16.38	20.03	
No	82.02	80.68	83.58	83.62	79.97	
BMI (kg/m^2^)	29.08 ± 5.67	28.60 ± 5.01	29.55 ± 5.72	29.22 ± 5.70	28.91 ± 6.13	0.006
Waist circumference (cm)	103.98 ± 14.36	102.47 ± 13.23	104.72 ± 13.73	104.35 ± 14.61	104.30 ± 15.59	0.010
Triglyceride (mg/dL)	148.24 ± 117.24	154.60 ± 145.97	148.30 ± 115.51	149.73 ± 108.23	139.88 ± 90.23	0.341
Total cholesterol (mg/dL)	197.98 ± 42.32	194.13 ± 41.96	202.21 ± 42.85	200.23 ± 42.02	194.93 ± 41.90	<0.001
LDL‐C (mg/dL)	118.32 ± 35.24	114.22 ± 34.07	122.48 ± 34.98	117.72 ± 35.20	119.14 ± 36.23	0.011
HDL‐C (mg/dL)	47.52 ± 14.49	48.12 ± 16.19	46.50 ± 13.87	47.03 ± 13.28	48.51 ± 14.58	0.015
Vitamin D (nmol/L)	67.51 ± 22.73	65.38 ± 22.17	68.30 ± 24.69	68.00 ± 21.04	68.23 ± 22.83	0.034
CRP (mg/dL)	0.36 ± 0.83	0.20 ± 0.29	0.27 ± 0.44	0.31 ± 0.62	0.66 ± 1.41	<0.001
tPSA (ng/mL)	1.50 ± 2.36	1.26 ± 1.70	1.42 ± 1.87	1.40 ± 1.84	1.91 ± 3.52	<0.001
fPSA (ng/mL)	0.37 ± 0.45	0.34 ± 0.37	0.36 ± 0.34	0.36 ± 0.36	0.43 ± 0.65	0.001
f/t PSA ratio (%)	30.71 ± 12.32	33.11 ± 12.78	30.93 ± 12.16	30.41 ± 11.90	28.52 ± 12.07	<0.001
**High PCa risk, (%)**						<0.001
Yes	4.96	3.49	4.15	3.54	8.73	
No	95.04	96.51	95.85	96.46	91.27	

*Note*. Mean ± SD for continuous variables; (%) for categorical variables.

Abbreviations: CRP, C‐reactive protein; fPSA, free prostate‐specific antigen; f/t PSA ratio, free/total prostate‐specific antigen ratio; HDL‐C, high‐density lipoprotein cholesterol; LDL‐C, low‐density lipoprotein cholesterol; PCa, prostate cancer; Q, quartile; SII, systemic immune‐inflammation index; tPSA, total prostate‐specific antigen.

### 3.2 Association between SII and PCa risk

**Figure 2:**
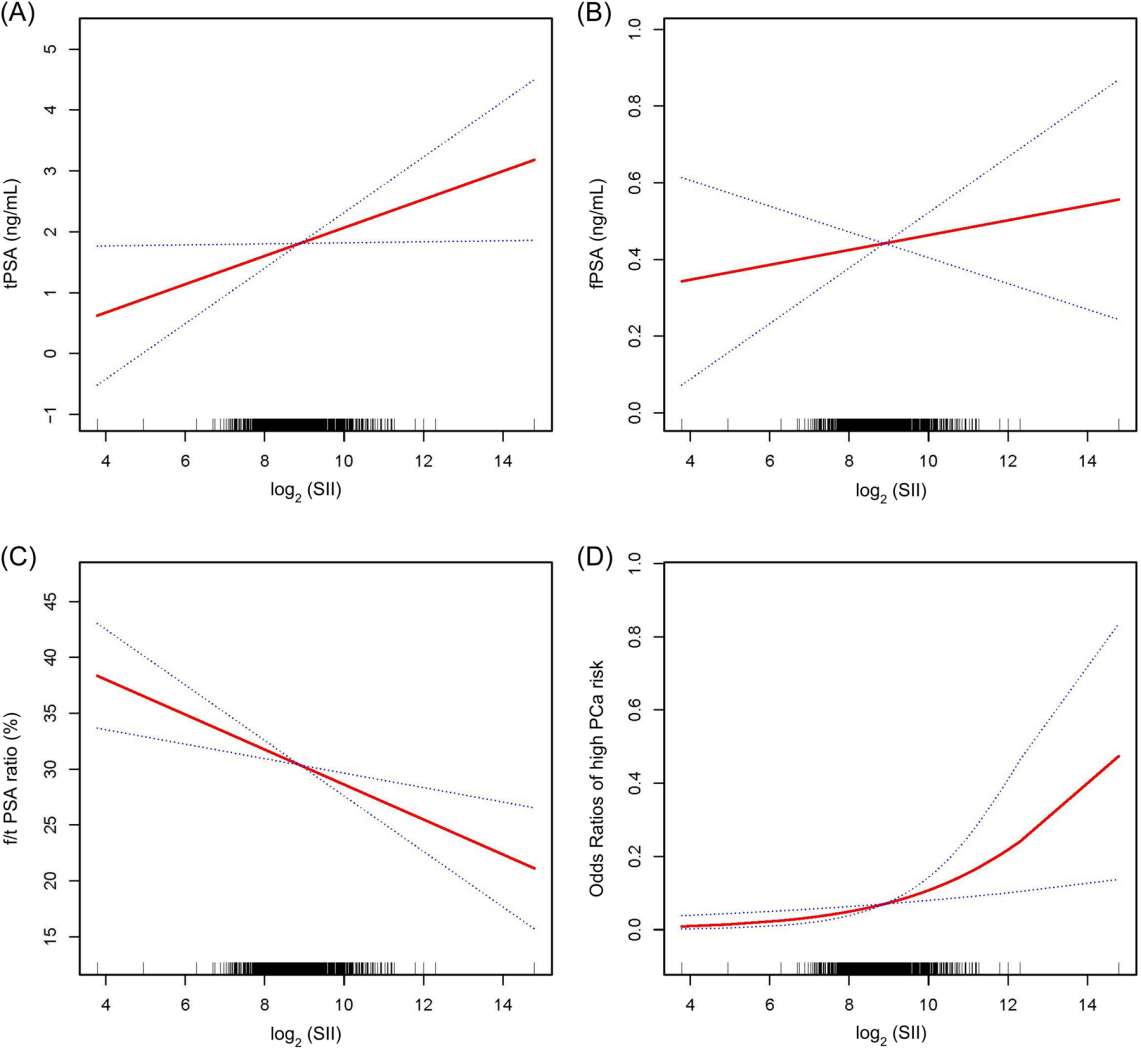
Nonlinear relationships between SII and PCa risk. The solid red line represents the smooth curve fitting between the variables, while the blue bands depict the 95% confidence interval from the fitting. (A). SII and tPSA; (B). SII and fPSA; (C). SII and f/t PSA ratio; (D). SII and high risk for PCa. fPSA, free prostate‐specific antigen; f/t PSA ratio, free/total prostate‐specific antigen ratio; PCa, prostate cancer; SII, systemic immune‐inflammation index; tPSA, total prostate‐specific antigen.

As delineated in Table 2, SII had a significant positive connection with both tPSA and risk for PCa, as well as a significant negative connection with f/t PSA ratio in all three models. When adjusted for all covariables, each unit increase of log_2_ (SII) was associated with a 0.22 ng/mL increase in tPSA (β: 0.22, 95% confidence intervals [CI] 0.05–0.38), a 2.22% decline in f/t PSA ratio (β: −2.22, 95% CI −3.20 to −1.23), and a 52% increased odds of being at high risk for PCa (odds ratio [OR]: 1.52, 95% CI 1.13–2.04). Results of the trend test further confirmed that the aforementioned correlation remained statistically significant across log_2_ (SII) quartiles (all *P* for trend <0.01). People in the top quartile of log_2_ (SII) had a 0.55 ng/mL increase in tPSA (β: 0.55, 95% CI 0.19–0.90), a 4.39% decline in f/t PSA ratio (β: −4.39, 95% CI −6.50 to −2.27), and a 168% increased risk of falling into the high‐risk category for PCa (OR: 2.68, 95% CI 1.32–5.46) compared to those in the bottom quartile. Although we did not find a significant relationship between SII and fPSA in either the partially or fully adjusted model, a slight upward trend in fPSA was observed along with the increasement of log_2_ (SII). Additionally, results of smoothing curve fitting and generalized additive model not only demonstrated the nonlinear positive correlation between SII with tPSA level and high risk for PCa, but also the nonlinear negative correlation between SII and f/t PSA ratio (Figure 2).

**Table 2:** Associations between systemic immune‐inflammation index and prostate cancer risk.

PCa risk	Model 1	Model 2	Model 3
**tPSA [β (95% CI)]**	0.26 (0.15, 0.36)	0.21 (0.11, 0.31)	0.22 (0.05, 0.38)
**Quartiles of log**_**2**_ **(SII)**			
Quartile 1	0 (ref)	0 (ref)	0 (ref)
Quartile 2	0.16 (−0.07, 0.39)	0.20 (−0.02, 0.42)	0.08 (−0.25, 0.42)
Quartile 3	0.14 (−0.09, 0.36)	0.15 (−0.06, 0.37)	0.15 (−0.18, 0.48)
Quartile 4	0.65 (0.42, 0.88)	0.52 (0.30, 0.74)	0.55 (0.19, 0.90)
*P* for trend	<0.001	<0.001	0.003
**fPSA [β (95% CI)]**	0.03 (0.01, 0.05)	0.01 (−0.00, 0.03)	0.02 (−0.02, 0.05)
**Quartiles of log**_**2**_ **(SII)**			
Quartile 1	0 (ref)	0 (ref)	0 (ref)
Quartile 2	0.02 (−0.03, 0.06)	0.02 (−0.02, 0.06)	0.01 (−0.06, 0.09)
Quartile 3	0.01 (−0.03, 0.05)	0.01 (−0.03, 0.05)	0.01 (−0.06, 0.08)
Quartile 4	0.08 (0.04, 0.12)	0.05 (0.00, 0.09)	0.06 (−0.02, 0.14)
*P* for trend	<0.001	0.051	0.172
**f/t PSA ratio [β (95% CI)]**	−2.14 (−2.68, −1.59)	−2.22 (−2.77, −1.67)	−2.22 (−3.20, −1.23)
**Quartiles of log**_**2**_ **(SII)**			
Quartile 1	0 (ref)	0 (ref)	0 (ref)
Quartile 2	−2.19 (−3.38, −1.00)	−2.38 (−3.57, −1.19)	−0.93 (−2.94, 1.07)
Quartile 3	−2.71 (−3.87, −1.54)	−2.98 (−4.15, −1.81)	−2.27 (−4.26, −0.29)
Quartile 4	−4.59 (−5.78, −3.40)	−4.66 (−5.86, −3.47)	−4.39 (−6.50, −2.27)
*P* for trend	<0.001	<0.001	<0.001
**High PCa risk [OR (95% CI)]**	1.34 (1.14, 1.57)	1.27 (1.08, 1.49)	1.52 (1.13, 2.04)
**Quartiles of log**_**2**_ **(SII)**			
Quartile 1	1 (ref)	1 (ref)	1 (ref)
Quartile 2	1.11 (0.74, 1.67)	1.21 (0.79, 1.84)	1.06 (0.48, 2.34)
Quartile 3	1.07 (0.71, 1.61)	1.09 (0.71, 1.67)	1.92 (0.93, 3.97)
Quartile 4	2.00 (1.38, 2.89)	1.86 (1.27, 2.74)	2.68 (1.32, 5.46)
*P* for trend	<0.001	0.002	0.002

*Note*. Model 1: no covariates were adjusted. Model 2: age and race were adjusted. Model 3: age, race, education level, poverty, living status, smoking status, drinking status, work activity, physical activity, hypertension, diabetes, BMI, waist circumference, triglyceride, total cholesterol, LDL‐C, HDL‐C, vitamin D, and CRP were adjusted.

Abbreviations: BMI, body mass index; CRP, C‐reactive protein; fPSA, free prostate‐specific antigen; f/t PSA ratio, free/total prostate‐specific antigen ratio; HDL‐C, high‐density lipoprotein cholesterol; LDL‐C, low‐density lipoprotein cholesterol; PCa, prostate cancer; SII: systemic immune‐inflammation index; tPSA, total prostate‐specific antigen.

### 3.3 Subgroup analyses

As delineated in Table 3, we conducted subgroup analyses and interaction tests, stratified by age, race, BMI, living status, smoking status, drinking status, and physical activity. The aim was to scrutinize the constancy of the relationship between the SII and PCa risk across diverse population segments and to identify potential distinctions within these population subsets. Our findings revealed no statistically significant discrepancies among the various subgroups, signifying that none of the variables materially altered the correlation between SII and PCa risk (all *P*‐values for interaction >0.05).

**Table 3:** Subgroup analyses of the relationship between systemic immune‐inflammation index and prostate cancer risk.

Subgroup	tPSA [β (95%CI)]	fPSA [β (95%CI)]	f/t PSA ratio [β (95% CI)]	High PCa risk [OR (95% CI)]
**Age**				
< 60 years	0.12 (−0.10, 0.34)	0.01 (−0.03, 0.06)	−1.91 (−3.21, −0.61)	0.71 (0.25, 1.98)
≥ 60 years	0.35 (0.10, 0.61)	0.03 (−0.03, 0.08)	−2.52 (−4.03, −1.02)	1.68 (1.23, 2.30)
*P* for interaction	0.161	0.774	0.539	0.114
**Race/ethnicity**				
Non‐Hispanic White	0.25 (0.06, 0.44)	0.03 (−0.02, 0.07)	−2.91 (−4.06, −1.76)	1.40 (0.92, 2.13)
Non‐Hispanic Black	−0.16 (−0.69, 0.36)	−0.06 (−0.18, 0.05)	0.15 (−3.05, 3.35)	1.20 (0.57, 2.54)
Other races	0.27 (−0.15, 0.69)	0.02 (−0.08, 0.11)	−1.95 (−4.60, 0.70)	2.11 (1.22, 3.65)
*P* for interaction	0.319	0.329	0.176	0.396
**BMI**				
< 24.9 kg/m^2^	0.20 (−0.09, 0.50)	0.04 (−0.02, 0.11)	−1.84 (−3.45, −0.24)	1.49 (0.88, 2.51)
25–29.9 kg/m^2^	0.21 (−0.05, 0.46)	0.01 (−0.05, 0.06)	−1.01 (−2.41, 0.40)	1.45 (0.94, 2.24)
≥ 30 kg/m^2^	0.25 (−0.05, 0.55)	0.03 (−0.04, 0.10)	−1.67 (−3.42, 0.08)	1.90 (1.02, 3.54)
*P* for interaction	0.970	0.683	0.704	0.763
**Living Status, (%)**				
With partners	0.26 (0.06, 0.46)	0.03 (−0.01, 0.08)	−2.21 (−3.36, −1.06)	1.66 (1.14, 2.41)
Alone	0.18 (−0.15, 0.50)	−0.02 (−0.08, 0.05)	−3.04 (−4.92, −1.16)	1.42 (0.78, 2.60)
*P* for interaction	0.663	0.183	0.450	0.664
**Smoking status, (%)**				
Nonsmoker	0.26 (0.00, 0.52)	0.02 (−0.04, 0.08)	−2.31 (−3.80, −0.83)	1.66 (1.07, 2.55)
Former smoker	0.26 (−0.00, 0.53)	0.02 (−0.04, 0.08)	−2.21 (−3.77, −0.66)	1.73 (1.10, 2.73)
Current smoker	−0.00 (−0.39, 0.39)	−0.00 (−0.09, 0.09)	−1.79 (−3.95, 0.37)	0.16 (0.02, 1.62)
*P* for interaction	0.466	0.915	0.917	0.077
**Drinking status, (%)**				
≥12 drinks/year	0.18 (−0.00, 0.36)	0.01 (−0.03, 0.05)	−2.14 (−3.21, −1.06)	1.41 (1.01, 1.98)
<12 drinks/year	0.45 (0.06, 0.84)	0.06 (−0.02, 0.15)	−3.53 (−6.04, −1.02)	2.03 (1.05, 3.95)
*P* for interaction	0.192	0.248	0.308	0.336
**Physical activity, (%)**				
Inactive	0.19 (−0.05, 0.43)	0.03 (−0.03, 0.08)	−1.73 (−3.14, −0.31)	1.55 (1.05, 2.29)
Moderate	0.33 (0.04, 0.62)	0.01 (−0.06, 0.07)	−2.82 (−4.50, −1.13)	1.69 (0.97, 2.93)
Vigorous	0.10 (−0.29, 0.49)	0.03 (−0.06, 0.12)	−2.20 (−4.52, 0.11)	0.90 (0.15, 5.21)
*P* for interaction	0.601	0.885	0.610	0.796

*Note*. Age, race, education level, poverty, living status, smoking status, drinking status, work activity, physical activity, hypertension, diabetes, BMI, waist circumference, triglyceride, total cholesterol, LDL‐C, HDL‐C, vitamin D, and CRP were adjusted.

Abbreviations: BMI, body mass index; CRP, C‐reactive protein; fPSA, free prostate‐specific antigen; f/t PSA ratio, free/total prostate‐specific antigen ratio; HDL‐C, high‐density lipoprotein cholesterol; LDL‐C, low‐density lipoprotein cholesterol; PCa, prostate cancer; SII, systemic immune‐inflammation index; tPSA, total prostate‐specific antigen.

## 4 DISCUSSION

Within our cross‐sectional study comprising 3359 male participants aged ≥40 years, a substantial association between SII and PCa risk was evident. In this cohort, SII exhibited a positive correlation with tPSA levels and the risk for PCa, while displaying a negative relationship with the f/t PSA ratio. Upon conducting subgroup analyses, these associations remained consistent across each stratum. Our findings suggest that SII may hold significant clinical potential in the realm of PCa risk prediction.

A mounting body of evidence has illuminated the role of systemic inflammatory responses in the initiation and progression of PCa.^6^ Consequently, various hematological indicators, including the PLR, NLR, and LMR have been proposed as indicative PCa markers.^9^ As a novel and composite index combining platelet, neutrophil, and lymphocyte counts, SII was found to be more objective than above‐mentioned prognostic indicators in PCa patients.^29^ The value of SII in guiding clinicians select the best treatment for metastatic castration‐refractory prostate cancer (mCRPC) has been reported in clinical studies.^30^ However, past epidemiological studies about the relationship between SII and PCa is scarce and with unconformable results. A retrospective case‐control study, which included 396 participants, concluded that SII was a powerful tool for the detection of localized PCa,^16^ whereas another prospective cohort study including 1223 Chilean men denied the diagnostic value of SII in PCa.^31^ These discrepancies may stem from variations in participant characteristics, sample size, or covariates. Two other cross‐sectional studies utilizing the NHANES cohort revealed a positive correlation between SII and the prevalence of PCa.^32^, ^33^ However, the study by Luo et al. failed to achieve statistical significance,^32^ leading to inconsistent conclusions between the two. Our study is the first investigation of the relationship between SII and PCa risk in middle‐aged and older US men with no prior history of PCa, distinctly diverging from these two studies that focused on PCa prevalence as the outcome measure. Furthermore, this study integrated physical activity, acknowledged as a significant risk factor for PCa,^34^ into the covariates, highlighting one of its strengths. In all, we identified a significant positive correlation between SII and PCa risk within the US population.

The precise mechanisms underpinning the observed positive association between SII and PCa risk remain enigmatic. Conducted research have proved that immune cells, such as lymphocytes, platelets, and neutrophils, in the tumor microenvironment (TME) affect tumor growth and metastasis.^35^ The association between immune cells and PCa was also investigated. Platelets can enhance the invasive potential of prostate cancer stem‐like cells through tumor cell‐induced platelet aggregation, thereby promoting progression of PCa.^36^ Additionally, platelet‐derived proangiogenic cytokines facilitate PCa tumor growth by stimulating abnormal angiogenesis and contribute to PCa bone metastasis by inhibiting osteoclastogenesis and increasing tumor‐induced bone formation.^37^, ^38^ Notably, in a clinical trial, the administration of antiplatelet therapy was linked to improved freedom from biochemical failure in patients undergoing primary radiotherapy for nonmetastatic PCa.^39^ Tumor‐infiltrating neutrophils represent a major population in the immunosuppressive TME of PCa, which drive tumor progression and treatment resistance.^40^ Nicolò Bancaro et al. reported the identification of senescent‐like neutrophils, which persist in the TME of PCa and own strong immunosuppression and tumor‐promoting ability.^41^ In a pilot study concerning immune checkpoint therapy in PCa, a transcriptional upregulation in neutrophil immune subset signature was identified within prostate bone microenvironment.^42^ In contrast to neutrophils, sparse infiltration of lymphocytes is another feature of prostate TME.^43^ Promoting the activation and infiltration of cytotoxic T lymphocytes by targeting the chromatin effector (pygopus family PHD finger 2) has been regarded as a potential path to overcome immunotherapy resistance in PCa.^44^ Besides the well‐known function of lymphocytes in mechanisms of immunotherapy resistance, lower counts of certain lymphocytes including subsets such as NK and CD4 + T cells were proven to be prognostically unfavorable for PCa patients.^45^ In our study, a high SII, which means high platelet or neutrophil counts and/or low lymphocyte counts, shows association with high PCa risk in middle‐aged and older adults without a PCa history and this positive association may follow mechanisms mentioned above.

Older age and African ancestry are known to be the two most important risk factors for PCa.^46^ Obesity is also associated with adverse prognosis in PCa.^47^ Our fully adjusted model included age, race, and obesity indicators like BMI and WC as covariates. Furthermore, results from our subgroup analysis stated the positive correlation between SII and the risk for PCa remained robust regardless of age, race, BMI, living status, smoking status, drinking status, and physical activity, consistent with previous research concentrated on SII and risk for developing other solid tumors.^48^

This study's primary contribution lies in the novel identification of an association between the SII and PCa risk among men in the United States who have no prior history of PCa. SII emerges as a potential indicator for assessing PCa risk within this specific population. Furthermore, the diagnostic value of SII for PCa remains subject to further scrutiny, although SII offers a robust assessment of the systemic immune and inflammatory status within the human body. Comparative investigations and longitudinal prospective research are imperative to ascertain the diagnostic accuracy and clinical utility of SII in relation to other established measurements. SII may function as a valuable complementary tool in the diagnostic arsenal of PCa through furnishing supplementary insights into the inflammatory status of patients. Given the firmly established association between systemic inflammatory responses and cancer, integrating SII into the diagnostic framework could aid in identifying individuals at elevated risk who may benefit from further evaluation.

Despite sparse clinical validation evidence, artificial intelligence (AI) and deep learning model have significantly advanced in diagnosing, predicting survival outcomes, and assessing treatment responses in PCa.^49^ A recent deep learning model incorporating tPSA, fPSA, [‐2]proPSA, PSA density, and age exhibited excellent performance in identifying high‐grade PCa, achieving an optimized sensitivity of up to 86% and a specificity of up to 89%.^50^ Our study found a close association between the SII and the risk for PCa. Consequently, a deep learning model based on SII may provide substantial support for diagnosing PCa, addressing the low specificity of current PSA screening. This offers a promising direction for future research.

This study encompasses several notable strengths. Firstly, we harnessed the rich and nationally representative NHANES database, acquired through a standardized protocol, as our data source. Secondly, we implemented rigorous adjustments for potential confounding factors, bolstering the reliability of our findings. For instance, previous research has indicated the influence of lipid metabolism on PSA concentrations, potentially introducing bias in PCa screening.^51^ Thus, to make our analysis more reliable, HDL‐C, LDL‐C, total cholesterol, and triglyceride were selected as covariates in our fully adjusted model (model 3). Thirdly, we conducted subgroup analyses to corroborate the association between SII and PCa risk across diverse populations, thereby reinforcing the statistical validity of our results. However, the limitations of our research cannot be ignored. First, due to the essence of the NHANES as a cross‐sectional survey which lacks follow‐up data on respondents, we could not draw any conclusions about causality. Therefore, large‐sample prospective studies are still required to illuminate the cause‐and‐effect relationship. Second, although we tried to minimize the potential bias in estimating this positive association, unmeasured confounders were challenging to include due to database limitations. For instance, low testosterone levels were found to be significantly associated with reduced biochemical recurrence‐free survival in PCa patients.^52^ However, NHANES did not conduct testosterone testing during the 2007–2010 period, preventing its inclusion as a covariate in this study. Lastly, our findings were geographically restricted because the NHANES database is limited to the US population.

## 5 CONCLUSION

This nationally representative study suggested that SII is independently and positively associated with tPSA levels and the risk for PCa, as well as independently and negatively associated with f/t PSA ratio among middle‐aged and older US adults. These results indicate that elevated systemic inflammatory status might be linked to increased odds of being at high risk for PCa. Upon further validation through longitudinal prospective studies, the SII could potentially emerge as a valuable adjunct to PCa screening to predict a positive biopsy result.

## AUTHOR CONTRIBUTIONS

**Wentao Yao:** Conceptualization; data curation; formal analysis; methodology; software; writing—original draft preparation; funding acquisition. **Jiacheng Wu:** Data curation; formal analysis; writing—original draft preparation. **Ying Kong:** Formal analysis. **Feng Xu:** Methodology. **Yinyi Zhou:** Software. **Qing Sun:** Visualization. **Qingqing Gao:** Visualization. **Zhenyu Cai:** Supervision; writing—review and editing. **Chendi Yang:** Conceptualization; supervision. **Yuhua Huang:** conceptualization; supervision; writing—review and editing; funding acquisition. All the authors have meticulously reviewed and unanimously concurred with the final published version of the manuscript.

## CONFLICT OF INTEREST STATEMENT

The authors declare no conflict of interest.

## ETHICS STATEMENT

This study used 2007–2010 NHANES data approved by the NCHS Research Ethics Review Board (continuation of Protocol #2005‐06, ERB). Written informed consent was provided by all NHANES participants.

## Supporting information


**Figure S1** A. The distribution of SII; B. The distribution of log_2_‐transformed SII. Abbreviation: SII, systemic immune‐inflammation index.

## Data Availability

Data analyzed during this study are publicly available online (https://wwwn.cdc.gov/nchs/nhanes/, accessed on 28 November 2023).
