# Supplementary figures and images for: Associations of systemic immune‐inflammation index with high risk for prostate cancer in middle‐aged and older US males: A population‐based study
Source: Immun Inflamm Dis. 2024 Jun 24;12(6):e1327. doi: 10.1002/iid3.1327 (PMC11194977; doi:10.1002/iid3.1327)

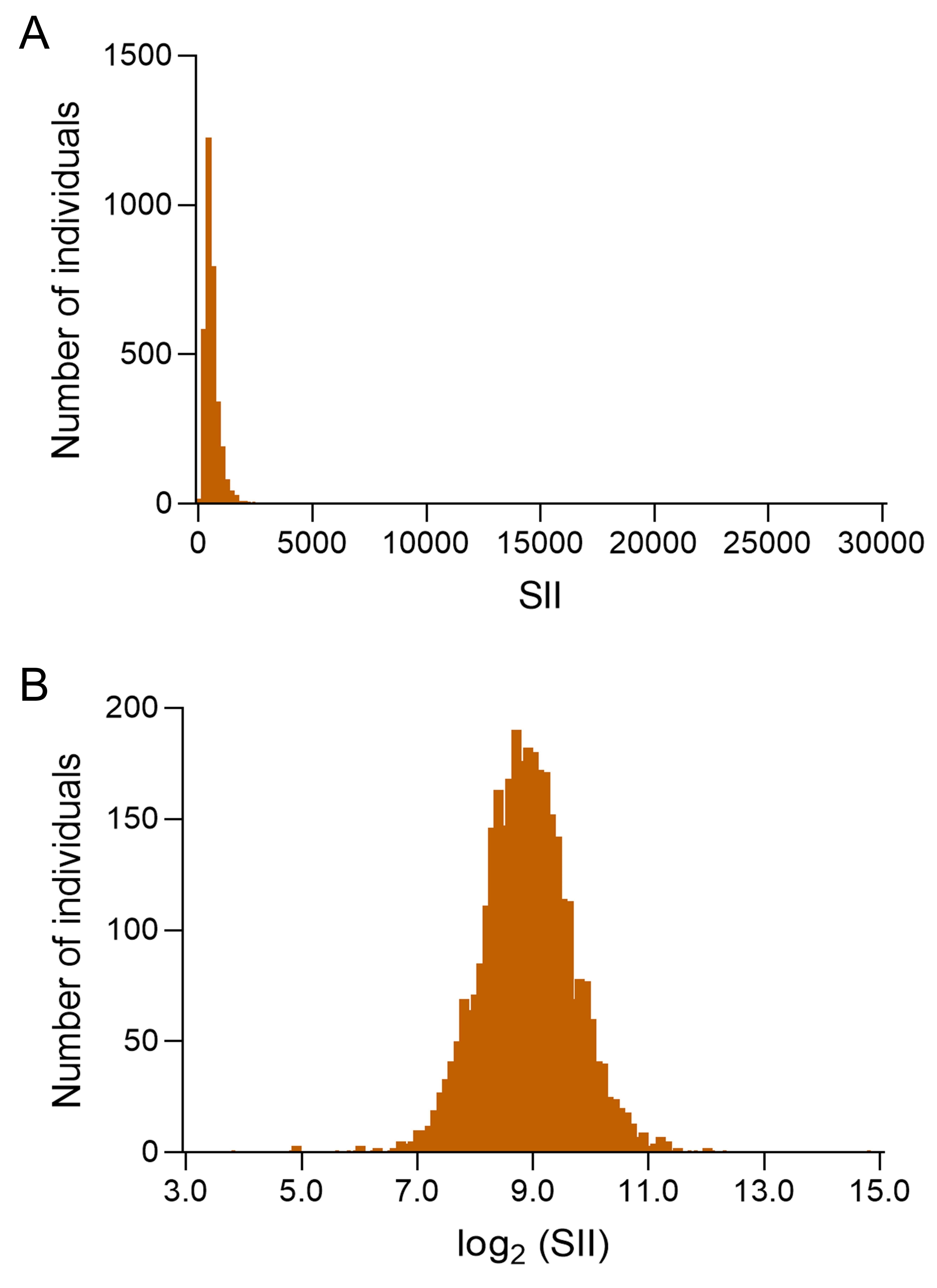

Supplement: Supplementary file 1 — Figure S1 A. The distribution of SII; B. The distribution of log2‐transformed SII. Abbreviation: SII, systemic immune‐inflammation index. [file IID3-12-e1327-s001.jpg]
